# Assessment of sphenoid sinus related anatomic variations with computed tomography

**DOI:** 10.11604/pamj.2017.27.109.7391

**Published:** 2017-06-13

**Authors:** Figen Tunalı Turkdogan, Kenan Ahmet Turkdogan, Murat Dogan, Mehmet Haydar Atalar

**Affiliations:** 1 Aydın Atatürk State Hospital, Department of Radiology, Aydın, Turkey; 2 Adnan Menderes Universty, Department of Emergency Medicine, Aydın, Turkey; 3 Adnan Menderes Universty, Department of Otorhinolaryngology, Aydın, Turkey; 4 Cumhuriyet University, Department of Radiology, Sivas, Turkey

**Keywords:** Sphenoid sinus, anatomic variations, paranasal sinuses computed tomography

## Abstract

**Introduction:**

Frequent and broad application of endoscopic sinus surgery (ESS) in parallel with experience makes it imperative to know the anatomy and the existing pathology very well before surgery. This study examines the association between anomalies in the sphenoid sinus area in paranasal sinuses computed tomography (PNS-CT) and pathological findings and determines variations of sphenoid sinus.

**Methods:**

A total of 200 cases (100 women, 100 men) who had PNS-CT in the emergency and radiology polyclinics within the period of one year were included in this study. Bone tissue anomalies and soft tissue pathologies were assessed in the CT.

**Results:**

Pterygoid process was found in 36.75% of our cases, anterior clinoid pneumatization was found in 21.25%; vidian canal in 34.25%, foramen rotundum in 17.5% and ICA in 12.75% had protrusion into the sphenoid sinus; 8.25% were found to have onodi cell, 11.25% were found to have multiple septation, 16.75% were found to have mucosal thickening and 2.5% were found to have retention cyst.

**Conclusion:**

The importance of PNS-CT in terms of determining anatomic variations before ESC and predicting possible complications during surgery has been emphasized once more. In our study, as sphenoid sinus pneumatization increased, the projection of neighbouring vein and nerve structures into the sinus was found to increase as well.

## Introduction

Endoscopic sinus surgery (ESS), which gains importance each passing day, is a method safely used in the treatment of paranasal sinus diseases. Frequent and broad application of ESS in parallel with experience makes it imperative to know the anatomy and the existing pathology very well before surgery. Pathology of sphenoid sinus and the neighbouring structures is an endoscopic surgical method frequently used by otolaryngologists and skull base surgeons. Thus, anatomic assessment of each patient before surgery is important in preventing intraoperative complications [1]. Since the progress of optical nerve and internal carotid artery is important, preoperative imaging has become a standard procedure. Axial and coronal tomography imaging is required for the correct presentation of anterior skull base, lateral wall and sinuses [2]. Today, computed tomography is a golden standard procedure in the imaging of sinuses [3]. As of its localization, sphenoid sinus acts as a corridor for surgeons in reaching neighbouring structures [4]. However, during surgery, serious complications may occur as a result of injuries of vital neighbouring structures such as internal carotid artery and optical nerves [5, 6].

Thus, an extensive knowledge of anatomic variables will certainly decrease surgical risks [7]. In transsphenoidal and skull base surgery, presenting sphenoid sinus ostium is an important step and guide [8, 9]. The spheonid sinüs is deriving from cephalic mesoderm (orbito-sphenoid part) and neural crest (basis-post sphenoid part) which placed in the mid part of the skull [10]. Understanding the sphenoid sinus anatomy as a whole is a must for successful sinus surgery [11]. This sinüs can be detected earlier ages (<2 years) on PNS-CT scan and it reaches its mature form within 14 years [12]. Sphenoid sinus has a changeable anatomic structure [11]. Despite the normal direction of pneumatization is inferoposterolateral way, the direction of pneumatization can shows quite variances in each human subject [12]. Patient specific pneumatization, different numbers and positions of septa affect the attitudes of surgeons towards skull base and paracellular space [11]. Although isolated sphenoid sinus pathologies are rate entities, they are still significant since delays in their diagnosis and treatment may cause serious problems. This significance results from the fact that sphenoid sinus is adjacent to vital structures such as optical nerve, internal carotid artery, cavernous sinus and skull base and from the fact that its anatomy is quite varied. As well as experience, defining the anatomic reference points in the surgical technique used and understanding the variations are also indispensable for highest success and lowest complication. With pre-operative imaging, important information specific for each patient is obtained; besides a road map is determined for safe surgery and approaches towards bones and soft tissues are determined [13]. This study examines the association between anomalies in the sphenoid sinus area in paranasal sinus tomographies (PNS-CT) and pathological findings and determines variations of sphenoid sinus. Bone tissue anomalies (pterygoid process and anterior clinoid process pneumatization, vidian canal and foramen rotundum bone canal protrusion, onodi cell, multiple septation) and soft tissue pathologies (mucosal thickening, polyp, cyst) were examined.

## Methods

### Patients

A total of 200 cases (100 women, 100 men) who had PNS-CT in the emergency and radiology polyclinics within the period of one year were included in this retrospective study. Bone tissue anomalies such as pterygoid process and anterior clinoid process pneumatization, vidian canal and foramen rotundum bone canal protrusion, onodi cell, multiple septation and soft tissue pathologies such as mucosal thickening, polyp and retention cyst were examined in the PNS-CTs.

### Statistical analysis

The comparisons of multiple variances were applied with using one-way ANOVA test and significant parameters were analysed with post hoc test. Mann-Whitney U-test was used to determine the differences between two groups. SPSS 15.0 software statistical analyse program was used to analysing of each statistical test. The p value <0.05 was expressed as significant.

## Results

**Figure 1:**
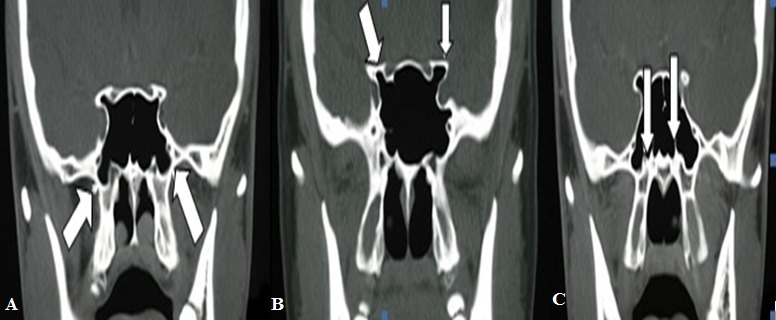
(A) bilateral Pterygoid process pneumatization in coronal PNS-CT; (B) bilateral anterior clinoid process pneumatization in coronal PNS-CT, (C) vidian canal protrusion in coronal PNS-CT

A total of 200 cases-100 men and 100 women (between the ages 18 and 70)- underwent bilateral examination as 400 sides. Different pathologies were assessed as existent, nonexistent, unilateral and bilateral. Pterygoid process pneumatization (Figure 1 A) was observed in a total of 147 sides, 106 of which were bilateral (53 cases) and 41 of which were unilateral (21 left, 20 right). In women, left, right and bilateral pterygoid process pneumatization was found as 7,12,25, respectively while in men, it was found as 14,8,28, respectively. The groups were similar in terms of gender. Unilateral pneumatization (0.433) and bilateral pneumatization (0.975) were detected as insignificant. Anterior clinoid process pneumatization (Figure 1 B) was observed in a total of 85 sides, 58 of which were bilateral (29 cases) and 27 of which were unilateral (11 left, 16 right). In women, left, right and bilateral anterior clinoid process pneumatization was found as 7,7,15, respectively while in men, it was found as 4,9,14, respectively. The groups were similar in terms of gender. Unilateral pneumatization (0.458) and bilateral pneumatization (0.405) were detected as insignificant. Vidian canal protrusion (Figure 1C) was observed in a total of 137 sides, 98 of which were bilateral (49 cases) and 39 of which were unilateral (20 left, 19 right). In women, left, right and bilateral vidian canal protrusion was found as 6,13,22, respectively while in men, it was found as 14,6,27, respectively. The groups were similar in terms of gender. Unilateral pneumatization (0.351) and bilateral pneumatization (0.908) were detected as insignificant.

**Figure 2:**
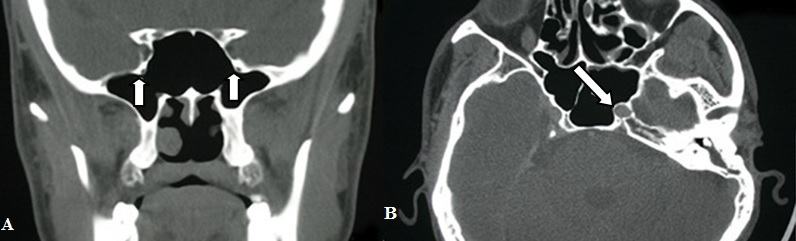
(A) bilateral foramen rotundum protrusion in coronal PNS-CT; (B) left ICA protrusion in axial PNS-CT

Foramen rotundum protrusion (Figure 2 A) was observed in a total of 70 sides, 38 of which were bilateral (19 cases) and 32 of which were unilateral (15 left, 17 right). In women, left, right and bilateral foramen rotundum protrusion was found as 8,8,11, respectively while in men, it was found as 7,9,8, respectively. The groups were similar in terms of gender. Unilateral pneumatization (0.788) and bilateral pneumatization (0.760) were detected as insignificant. ICA protrusion (Figure 2 B) was observed in a total of 51 sides, 26 of which were bilateral (13 cases) and 25 of which were unilateral (17 left, 8 right). In women, left, right and bilateral ICA protrusion was found as 8,3,7, respectively while in men, it was found as 9,5,6, respectively. The groups were similar in terms of gender. Unilateral pneumatization (0.482) and bilateral pneumatization (0.576) were detected as insignificant. Onodi cell was observed in a total of 33 cases, 17 of whom were men and 16 of whom were women. Mucosal thickening was observed in a total of 67 sides, 56 of which were bilateral (28 cases) and 11 of which were unilateral (2 left, 9 right). In women, left, right and bilateral mucosal thickening was found as 0,5,14, respectively while in men, it was found as 2,4,14, respectively. Multiple septation was observed in a total of 45 cases, 30 of whom were men and 15 of whom were women. Retention cyst was observed in a total of 10 sides, 8 of which were bilateral (4 cases) and 2 of which were unilateral (1 left, 1 right).

## Discussion

Although conventional radiographic examinations are used frequently and as the first step examination in practical application, they do not allow for the obvious imaging of ethmoid and sphenoid sinuses [14, 15]. When these are compared by CT, rates of pseudo positive and pseudo negative findings are known to be very high [15, 16]. In tomographic sections, bone anomalies and mucosal pathologies can be assessed together. It is known that although conventional graphies do not give enough information about osteomeatal complex, frontal recess, posterior ethmoid sinuses, ethmoid bulla, sphenoid sinus and sphenoethmoidal recess and anatomical variations, these can be shown in detail with coronal CT [14–17]. Today, in a great number of centers, coronal PNS-CT is used routinely for the diagnosis of chronic sinus diseases.

Since the levels of sphenoid sinus pneumatization vary to a great extent, they are classified as three types; conchal, presellar and sellar [18]. With surgical techniques that developed in addition to these, this classification was enlarged into four types; conchal, presellar, sellar and postsellar [12].

Sphenoid sinuses expand into the sphenoid bone back from posterior ethmoid cells and expand into the pterygoid laminas with the greater wings of the sphenoid bone [19]. As sphenoid sinus expands, carotid artery and optical nerve are reported to form a bulge on the sinus lateral wall in parallel with the amount of expansion [15]. In our study, in parallel with this view, the vein and nerve structures neighbouring the sinus were found to remain within the sinus as the sinus pneumatization increased. When this expansion continues to anterior clinoid processes, there occurs a risk of injury for the optical nerve during surgery [20, 21]. It is extremely important to have information before surgery on an area which shows so much variation. For the same reason, showing maximum attention to the sphenoid sinus walls during ESC is necessary to protect from possible complications.

In the literature, the incidence of clinoid pneumatization was reported as 13.3-16.0% (6.4-8.0% bilateral) [17], the rate of vidian canal protrusion on the sphenoid sinus base was reported as 7.5-13.3% (1% bilateral) [17, 22, 23] and the rate of pterygoid process pneumatization was reported as 15.5-43.6% (22.3% bilateral) [17, 22, 24]. Different sources have reported 4.8-22% bone defect on carotid artery [13, 20, 21], 4-12.9% projection of foramen rotundum into the sinus [22, 23], 1-32% incidence of septal cells [15] and 0-18% incidence of onodi cells [3, 22, 23, 25, 26]. In our study, the rate of anterior clinoid process pneumatization was found as 21.25%, while the rate of pterygoid lamina pneumatization was found as 36.75%; the rate of vidian canal in sphenoid sinus was 34.25%, the rate of foramen rotundum protrusion was 17.50% and the rate of ICA protrusion was 12.75%.

ICA protrusion was found to increase as anterior clinoid pneumatization increased. Different from the literature, there was too much anterior clinoid pneumatiziation, vidian canal and foramen rotundum protrusion and they were found to have a more frequent course with pterygoid lamina pneumatization. With the increase in pterygoid lamina pneumatization, an increase was found in the projection of vidian canal into the sinus. Anatomic variations are claimed to have a predisposing effect on the formation of sinus disease and mucosal disease is claimed to occur more in these [15]. While a statistically significant difference was found between the control group and the patient group in CT analysis in terms of mucosal pathologies, no significant difference was found for the bone structure [15, 17].

In literature, the presence of anterior clinoid and pterygoid process pneumatizations and onodi cells was reported to have no effect on the pathophysiology of sinus disease [15] and 4-39% of mucosal pathology was found in sphenoid sinuses in CT [15–17, 23].

In our study, 16.75% mucosal thickening was found in sphenoid sinus and no polyp was found. In line with the literature, bony anomaly and mucosal pathology were not found to progress together to a large extent.

## Conclusion

Today, having information about the anomalies and pathologies of the area before endoscopic surgery of sphenoid sinuses, skull base surgeries and lateral wall surgeries is very important to realize and protect the anatomic variations of structures which have vital importance around this area in terms of preventing the complications that may occur during surgery. In our study, in line with literature, a significant amount of sphenoid sinus anatomic variations was found and the importance of PNS-CT in determining these was emphasized once more.

### What is known about this topic

- Sphenoid sinus acts as a corridor for surgeons in reaching neighbouring structures;
- Sphenoid sinus is adjacent to vital structures such as optical nerve, internal carotid artery, cavernous sinus and skull base and from the fact that its anatomy is quite varied.

### What this study adds

- Sphenoid sinus anatomic variations is very important preventing to the complications;
- This area which have vital anatomic structure prevent surgical injury and decrease of morbidity.

## Competing interests

The authors declare no competing interest.
